# Chemoenzymatic Synthesis of Selegiline: An Imine Reductase-Catalyzed Approach

**DOI:** 10.3390/molecules29061328

**Published:** 2024-03-16

**Authors:** Yuliang Hu, Jinping Bao, Dongyu Tang, Shushan Gao, Fei Wang, Zhongtao Ding, Chengsen Cui

**Affiliations:** 1 College of Bioscience and Bioengineering, Jiangxi Agricultural University, Nanchang 330045, China; huyuliang98@163.com (Y.H.);; 2 Tianjin Institute of Industrial Biotechnology, Chinese Academy of Sciences, Tianjin 300308, China; 3 National Technology Innovation Center of Synthetic Biology, Tianjin 300308, China

**Keywords:** imine reductase, reductive amination, semi-rational design, homobenzylic amine, chemoenzymatic synthesis

## Abstract

(*R*)-Homobenzylic amines are key structural motifs present in (*R*)-selegiline, a drug indicated for the treatment of early-stage Parkinson’s disease. Herein, we report a new short chemoenzymatic approach (in 2 steps) towards the synthesis of (*R*)-selegiline via stereoselective biocatalytic reductive amination as the key step. The imine reductase IR36-M5 mutant showed high conversion (97%) and stereoselectivity (97%) toward the phenylacetone and propargyl amine substrates, offering valuable biocatalysts for synthesizing alkylated homobenzylic amines.

## 1. Introduction

**Figure 1:**
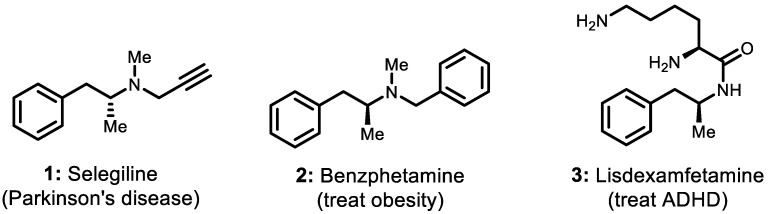
(*R*)-selegiline and related drug molecules.

Chiral amines, one category of the most commonly used chiral blocks in organic synthesis, are the crucial structural units of many pharmaceuticals, agrochemicals, fine chemicals, polymers, dyes, pigments, etc. [1]. Within the ample and diverse range of optically pure amines, homobenzylic amine derivatives are much sought-after structural motifs found in a wide variety of market-approved active pharmaceutical ingredients (APIs) and bioactive molecules. The former drug candidates of (*R*)-selegiline [2,3,4,5,6], benzphetamine [7,8], and lisdexamfetamine (Figure 1) are representative examples of these compounds [9]. (*R*)-Selegiline, as a pharmaceutical substance belonging to this category, is commonly used in clinical practice to reduce the symptoms of early-stage Parkinson’s disease. The pharmacological effect of (*R*)-selegiline is that it works as a selective irreversible MAO-B inhibitor slowing the breakdown of certain natural substances in the brain (e.g., dopamine, norepinephrine, and serotonin). (*R*)-Selegiline is usually used in combination with L-DOPA or carbidopa and can also be used for the treatment of depression and senile dementia [2,3,4,5,6].

**Figure 2:**
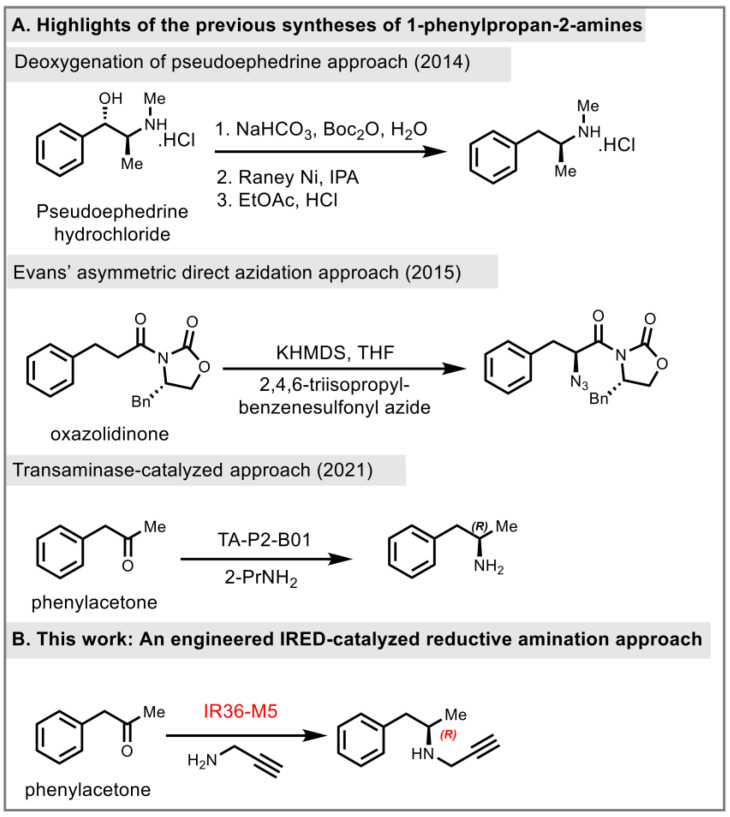
Prior synthesis and our synthetic strategy, 2014 [17], 2015 [18], 2021 [19].

Due to the highly potent biological activities of pharmaceutical substances belonging to this category, considerable efforts have been devoted to their asymmetric synthesis. The synthesis of (*R*)-selegiline has been reported through different strategies in recent years. Racemic approaches [10,11], chiral pool starting materials, asymmetric hydrogenation, classical/kinetic resolutions [12,13], regioselective aziridine ring openings with organocuprates [14], OsO_4_-catalyzed asymmetric dihydroxylations [15], and proline based functionalization of aldehydes [16] are the most reported synthetic strategies of (*R*)-selegiline. In 2014, Pramanik and co-workers reported a deoxygenation of the pseudoephedrine approach to prepare (*R*)-selegiline (Figure 2A) [17]. In the following year, Sudalai and co-workers employed Evans’ electrophilic azidation of chiral imide enolates as the key chiral-inducing step to synthesize (*R*)-selegiline in high enantioselectivity (97%, *R*) (Figure 2A) [18]. In 2021, Lavandera and co-workers employed amine transaminase (ATA) as a biocatalyst to synthesize (*R*)- and (*S*)-homobenzylic amines in 81% yield (>99%, *R*) and 80% yield (>99%, *S*), respectively [19]. However, it further required an extra 2 steps for alkylation of the primary amine for the synthesis of (*R*)-selegiline. Herein, we developed an engineered IR36-M5 catalyzed reductive amination approach to access the key intermediate (*R*)-desmethylselegiline, **1a** (Figure 2B). 

Organic synthesis using biocatalysts has become a research hotspot in green chemistry. Biocatalysis mediated by biological enzymes has the advantages that reactions are generally carried out at an ambient temperature, atmospheric pressure, and near-neutral conditions, with high catalytic efficiency, conversion, and stereo- and regioselectivity. Asymmetric reductive amination is one of the most direct and efficient strategies to synthesize chiral amines. Imine reductases (IREDs), one category of NADPH-dependent oxidoreductases, are of great interest because of their ability to catalyze asymmetric reductive amination of isostoichiometric prochiral ketones and amines to produce their corresponding amine products [20,21,22]. Turner and co-workers identified reductive aminases (RedAms), a specific sub-class of IREDs, which are capable of catalyzing reductive amination of ketones and amine in equal stoichiometric quantities [23]. In recent years, IREDs have exhibited their exceptional biocatalytic efficiency, chemo- and stereoselectivity under mild conditions, and have offered distinctive benefits in synthesizing alkylated chiral amines with a broad spectrum of ketones and amines [24,25,26,27,28,29,30,31,32,33,34]. Biocatalytic reductive amination using IREDs has led to commercial manufacturing. For example, engineered IREDs have been used in the large-scale production of the LSD1 inhibitor GSK2879552 [35] and the JAK1 inhibitor abrocitinib [36].

**Figure 3:**
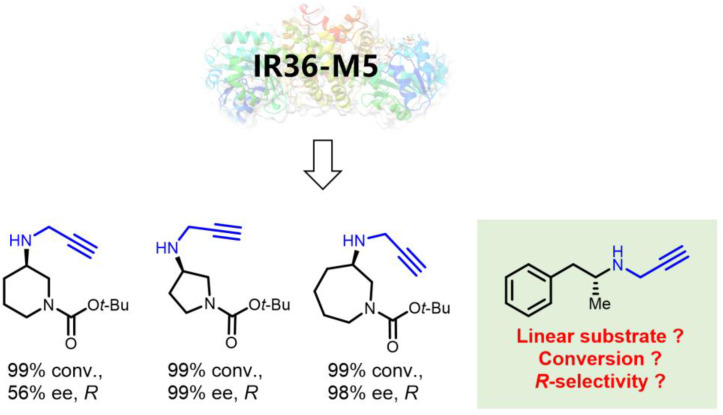
Our previous work on IR36-M5 catalyzed reactions towards cyclic substrates and proposed linear substrates.

In our previous research, we successfully developed the IR36-M5 mutant capable of catalyzing reactions involving piperidines and azepane alkylamines while also enhancing and reversing stereocenters [37,38]. Specifically, when utilizing propargylamine **a** as the donor, both pyrrolidinone and oxo-azepane exhibited outstanding *R*-stereoselectivity, achieving 98% and 99% ee values, respectively. However, when dealing with a piperidinone substrate, IR36-M5 displayed an excellent conversion rate of 99%, but the stereoselectivity was relatively lower at 56% (*R*) (Figure 3). These findings prompted us to explore the potential of this mutant further through a semi-rational design for synthesizing linear substrate, (*R*)-desmethylselegiline **1a**, a crucial intermediate in the (*R*)-selegiline molecule (Figure 3). When using phenylacetone **1** and propargylamine **a** as the ketone and amine donors, IRED variants exhibited limited stereoselectivity. In contrast, IR36-M5 displayed high stereoselectivity when applied to the phenylacetone **1** and propargylamine **a** substrate combination (Table 1). To enhance the catalytic capability of IRED for producing (*R*)-desmethylselegiline **1a**, we undertook a structure-guided semi-rational design approach, focusing on the residues that directly interacted with the ketone moiety. This effort resulted in five mutants generated through site saturation mutations at the identified hotspots, all of which exhibited improved stereoselectivity (98%, *R*) compared to M5 (97%, *R*) in the reductive amination of phenylacetone **1** and propargylamine **a**. The subsequent sample step is the reductive amination of **1a** with formaldehyde, leading to the successful synthesis of (*R*)-selegiline. We developed a new concise two-step chemoenzymatic synthetic pathway to (*R*)-selegiline with high stereoselectivity and high conversion (97%) in this study.

## 2. Results and Discussion

In order to produce the key intermediate (*R*)-desmethylselegiline **1a**, phenylacetone **1** and propargylamine **a** were chosen as the model substrates for biocatalytic reductive amination. In our previous work, we have developed the weakly active IR36 into a variant IR36-M5 with high performance for the synthesis of azacyclo-alkylamines, with a 4139-fold improvement in catalytic efficiency and ee values (>99%, *R*) [39]. Consequently, we opted to utilize IR36-M5 as our initial enzyme to test our model study. As depicted in Table 1, the IR36-M5 mutant displayed significant stereoselectivity, reaching 97% in the *R* configuration toward substrate **1**. We also tested several M5 mutants (M5-F260D, M5-F260E, M5-F260N, M5-W234L, and M5-W234I), which exhibited excellent stereoselective control in our pyrrolizidine alkylamine synthesis [38]. Most of these mutants exhibited relatively low to moderate enantioselectivity ranging from 52% to 85% (*R*) ee values. Disappointingly, the results show that these M5 mutants are not ideal biocatalysts to synthesize the key intermediate (*R*)-desmethylselegiline **1a**.

**Figure 4:**
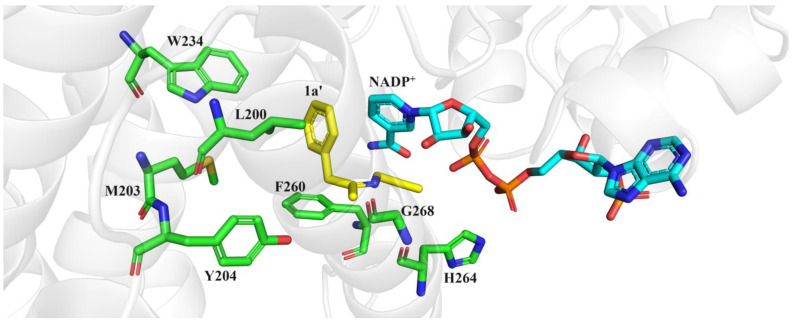
Docking the intermediate of reductive amination of **1** and **a** into the M5 (PDB: 7WNW) active cavity.

**Figure 5:**
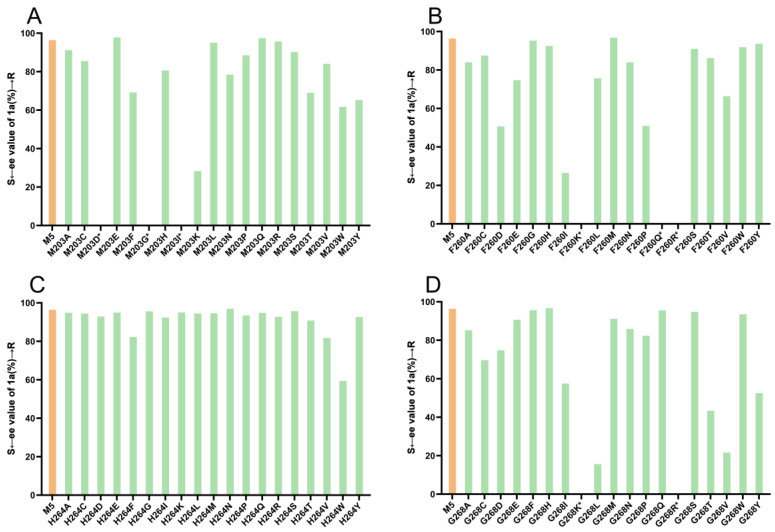
Enantioselectivity of M5 and its mutants in the reductive amination of **1** and **a**; (**A**–**D**) Enantioselectivity of M5 and its mutants at sites 203, 260, 264, and 268, respectively. * ee values were not determined because the mutants were almost inactive.

To identify potential ideal mutation sites, we conducted molecular docking of the imine intermediate **1a′** into the crystal structure of M5 (PDB: 7WNW) using Discovery Studio 2019. Our analysis revealed that seven amino acids, namely, F260, L200, M203, Y204, W234, H264, and G268, directly interacted with the phenylacetone **1** moiety (Figure 4). To further explore the effects of these sites on stereoselectivity, we conducted single-site saturation mutagenesis experiments on M5 at these seven amino acid positions. As illustrated in Figure 5 and Figures S1–S3, we observed that when the residue M203 was replaced by amino acids possessing smaller side chains, the resulting mutants displayed improved *R*-selectivity (Figure 5A and Table S2). However, when M203 was replaced by Asp, Gly, and Ile, the enzyme almost entirely lost its catalytic activity. Notably, the polar uncharged residue M203Q and the negatively charged residue M203E exhibited significant enhancements in *R*-selectivity, achieving up to 98% ee values.

In the positions of 264 (Figure 5C and Table S4) and 234 (Figure S3 and Table S8), most of the mutants displayed *R*-selectivity in moderate to good levels of *R*-selectivity. Notably, the mutant H264N exhibited significant enhancement in *R*-selectivity, achieving up to 98% ee value. In our previous study, F260 and W234 played pivotal roles in determining stereoselectivity when dealing with azacycloamines. In this case, when the residue at position F260 was replaced with a larger side-chain amino acid, the product exhibited high *R*-stereoselectivity (Figure 5B and Table S3). However, for positively charged and polar uncharged amino acids with larger side chains, the enzyme loses the catalytic activity. The result confirmed that the residue at position F260 played a pivotal role in biocatalytic reductive amination reactions. Similarly, in the case of G268, *R*-selectivity was preferred in asymmetric reductive amination involving the phenylacetone **1** substrate and the mutant G268H exhibited significant enhancement in R-selectivity, achieving up to 98% ee value. When the residues were replaced by Lys and Arg, both positively charged amino acids, the M5 variants lost their catalytic activity (Figure 5D and Table S5). Based on these findings, it is reasonable to speculate that the positions of 203, 243, 260, and 264 are crucial for the *R*-selectivity of IR36-M5, convincing the molecular docking predictions.

Regarding the position of L200, when we replaced the amino acid with His, Lys, Pro, Arg, Trp, or Tyr, we observed a complete loss of catalytic activity toward the model substrates (Figure S1 and Table S6). This means that most mutations in the L200 amino acid site lead to enzyme inactivation out of the seven amino acid sites. These results indicate that the L200 amino acid site is highly conserved. Conversely, when residue L200 was substituted with Met, Asn, Ile, and Val, we observed *R*-selectivity with good levels of ee values, reaching 95%, 89%, 86%, and 86%, respectively. This indicates that specific amino acid substitutions at position L200 can lead to improved *R*-selectivity while maintaining catalytic activity. The results are also consistent with the molecular docking prediction that residue L200 interacts very directly with the phenylacetone **1** moiety (Figure 4). At position 204, we also observed good *R*-selectivity with ee values reaching up to 94% (Figure S2 and Table S7).

Among all the single-site mutation variants in this study, five IR36-M5 mutants displayed 98% *R*-selectivity, specifically M203E, M203Q, F260M, H264N, and G268H, in comparison to IR36-M5 (97%, *R*). To assess the potential of IR36-M5 and its variants in the synthesis of (*R*)-desmethylselegiline **1a**, we further evaluated their conversion ratios for phenylacetone **1** (Table S9). Notably, IR36-M5 demonstrated excellent conversion, achieving 97% conversion (Table S9 Entry 1). However, the other five IR36-M5 mutants with 98% (*R*) ee values showed relatively poor to moderate conversions as follows: Entry 2, M203—34% conversion; Entry 3, M203Q—42% conversion; Entry 4, F260M—9% conversion; Entry 5, H264N—58% conversion; and Entry 6, G268H—65% conversion.

**Scheme 1:**
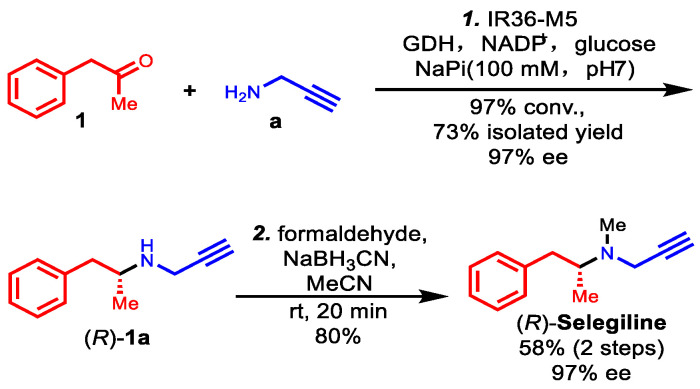
Chemoenzymatic synthesis of (*R*)-selegiline via IRED-catalyzed reductive amination.

Based on its remarkable catalytic activity and stereoselectivity, IR36-M5 was selected to facilitate the concise total synthesis of (*R*)-selegiline using a chemoenzymatic approach, as illustrated in Scheme 1. The process commenced with an enzymatic reductive amination of phenylacetone **1** and propargylamine **a**, employing M5 as the catalyst, resulting in the production of (*R*)-desmethylselegiline **1a** with a 73% isolated yield and 97% (*R*) ee. The ^1^H and ^13^C NMR data of **1a** produced using M5 are shown in Figures S5 and S6 and are in accordance with those reported in the literature [14]. This step was followed by a subsequent reductive amination with formaldehyde, leading to the successful synthesis of (*R*)-selegiline with an 80% yield. The ^1^H and ^13^C NMR data of (*R*)-selegiline synthesized via our IRED-catalyzed reductive amination are in accordance with those reported in the literature (Figures S7 and S8) [14]. This concise two-step chemoenzymatic synthesis of (*R*)-selegiline with high stereoselectivity and high conversion expands a new synthesis pathway for this Parkinson’s disease therapy, which is generally in use and may produce potential pharmacological value and economic benefits.

## 3. Materials and Methods

### 3.1. General

^1^H NMR spectra were recorded in CDCl_3_ (400 MHz). Residual solvent peaks are used as the internal reference; the signals at 7.26 ppm are set for ^1^H NMR spectra, taken in CDCl_3_. Silica gel plates pre-coated on glass were used for thin-layer chromatography using UV light, potassium permanganate solution, and heating as the visualizing methods. Silica gel was used for flash column chromatography with mixed Et_2_O or ethyl acetate (EtOAc) and *n*-hexane as the eluting solvents. HPLC analysis was performed on Waters 2695 using a C_18_ analytical column (Eclipse XDB-C_18_, 4.6 × 250 mm, 5 μm, Agilent, Santa Clara, CA, USA). LC-MS analysis was performed on Agilent 1100 with a mass spectrum detector (MSD) using an analytical column (Ultimate XB-C_18_, 2.1 × 100 mm, 3.0 μm, Welch, Shanghai, China). Chiral HPLC analysis was performed on Waters 2695 using chiral analytical columns (CHIRALPAK IG, etc., 4.6 × 250 mm, 5 μm, Daicel, Osaka, Japan). NMR spectra were recorded on a Bruker Avance 400 MHz spectrometer (Bruker, Bremen, Germany).

### 3.2. Materials

Commercially available chemicals and reagents, including ketone **1**, amine **a**, and NADP^+^, were purchased from Sigma-Aldrich (Poole, Dorset, UK), Macklin (Shanghai, China), or unless stated otherwise. All HPLC or LC-MS grade solvents, including acetonitrile, *n*-hexane, and ethanol, were purchased from Thermo Fisher Scientific (Waltham, MA, USA). Sodium phosphate buffer (0.1 M, pH 7.0) was prepared in-house. The *E. coli* BL21 (DE3) strain was purchased from Beijing Zoman Biotechnology Co., Ltd (Beijing, China).

### 3.3. Preparation of Standard Amines

#### 3.3.1. Reductive Amination Procedure for the Preparation of Racemic Amine (**1a**) Standards

A dry methanol (10 mL) solution of the phenylacetone **1** (0.75 mmol), the propargylamine **a** (1.12 mmol), and acetic acid (300 μL) under an N_2_ atm was stirred for 1.5 h at room temperature. The reaction was placed in an ice-water bath (0 °C), and sodium cyanoborohydride (140.5 mg, 2.24 mmol) was added. The reaction mixture was gradually warmed to room temperature and stirred overnight. The reaction progress was monitored by TLC and upon completion, it was quenched with saturated NaHCO_3_ solution (10 mL) and stirred for an additional 30 min. The mixture was extracted with EtOAc (3 × 10 mL) and the combined organic phase dried over Na_2_SO_4_ and concentrated under reduced pressure. Column chromatography on silica (silica gel column, gradient elute from pure PE-PE/EA, 90:10) affords the corresponding racemic **1a**. 

***rac*-1a:** ^1^H NMR (400 MHz, CDCl_3_) δ 7.36–7.33 (m, 2H), 7.28–7.24 (m, 3H), 3.52 (dd, *J* = 17.2, 2.5 Hz, 1H), 3.45 (dd, *J* = 17.2, 2.5 Hz, 1H), 3.21 (sextet, *J* = 6.5 Hz, 1H), 2.76 (dd, *J* = 13.4, 7.0 Hz, 1H), 2.67 (dd, *J* = 13.6, 6.5 Hz, 1H), 2.22 (t, *J* = 2.5 Hz, 1H), 1.11 (d, *J* = 6.2 Hz, 3H); ^13^C NMR (100 MHz, CDCl_3_) δ 139.2, 129.4, 129.4, 128.5, 128.5, 126.4, 82.1, 71.3, 52.6, 43.6, 35.7, 19.7. The ^1^H NMR data are in accordance with those reported in the literature [14].

#### 3.3.2. Reductive Amination Procedure for the Preparation of Chiral Amine Standards

Chiral amine standards were obtained from commercially available standards and were purchased from TRC (Toronto, ON, Canada).

#### 3.3.3. Enzymatic Synthesis Compound **1a**

At room temperature, a 10 mL enzymatic reaction is performed, which consists of 1 mM NADP^+^, cell-free extract (10 g/L wet cells weight, M5), phenylacetone **1** (30 mM, 40.2 mg), propargylamine **a** (60 mM, 37.7 mg, stock amine solution was adjusted to pH 7.0 with 1 M HCl), D-glucose (60 mM), 1 mg/mL glucose dehydrogenase GDH, and 10% DMSO in sodium phosphate buffer (100 mM, pH 7.0). After being shaken for 24 h at 30 °C with 200 rpm, the saturated Na_2_CO_3_ aqueous solution was added to prepare alkaline (pH 9~10), The mixture was extracted with EtOAc (3 × 10 mL), and the combined organic phase dried over Na_2_SO_4_ and concentrated under reduced pressure to yield crude product. The crude product was purified by silica gel column (gradient elute from pure PE-PE/EA, 90:10), and the pure product was colorless oil (37.9 mg, 73%) for the next step of synthesis. The NMR data are in accordance with ***rac*-1a** as described above. 

#### 3.3.4. The Synthesis of (*R*)-Selegiline

**Scheme 2:**
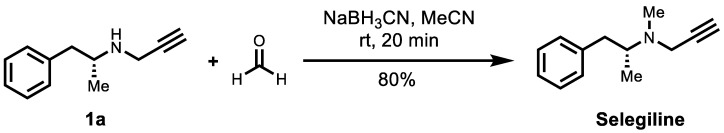
Synthesis of (*R*)-selegiline from **1a** through reductive amination with formaldehyde.

As shown in Scheme 2, 10 mg of compound **1a** in acetonitrile (0.2 mL) was added to 37% formaldehyde solution (29 mg) at 0 °C (ice-water bath). After 5 min, the resultant mixture was added to sodium cyanoborohydride (5.8 mg). After 2 min, the ice-water bath was removed and stirred at room temperature for 20 min. The mixture was diluted with 0.1 M potassium hydroxide and extracted with ether (3 × 10 mL). The organic phase was dried over Na_2_SO_4_, filtered, and concentrated in vacuo. The crude product was purified by silica gel column (gradient elute from Et_2_O/*n*-Hexane, 1:1), concentrated under cooling conditions (ice-water bath), and obtained the desired product as a colorless oil (8.6 mg, 80%).

**(*R*)-Selegiline:** ^1^H NMR (400 MHz, CDCl_3_) δ 7.37–7.33 (m, 2H), 7.28–7.24 (m, 3H), 3.50 (d, *J* = 2.4 Hz, 2H), 3.14–3.01 (m, 2H), 2.50 (s, 3H), 2.46 (dd, *J* = 12.7, 9.5 Hz, 1H), 2.31 (t, *J* = 2.5 Hz, 1H), 1.04 (d, *J* = 6.5 Hz, 3H); ^13^C NMR (100 MHz, CDCl_3_) δ 140.4, 129.4, 129.4, 128.4, 128.4, 126.1, 80.6, 72.6, 59.6, 43.3, 39.9, 37.6, 15.3. The ^1^H and ^13^C NMR data are in accordance with those reported in the literature [14].

### 3.4. Site-Saturation Mutagenesis Library Construction and Enantioselectivity Analysis of M5 Mutants

We constructed site-saturation mutagenesis libraries using the Fast Mutagenesis System (TransGen Biotech, Beijing, China). In detail, first, we replaced the codon of the mutation site with the NNK degeneracy codon (Supplementary Table S1). *E. coli* BL21 (DE3) was then transformed with pET28a-IR36-M5 generated by saturation mutagenesis. Colonies were picked up into 96-well deep-well plates containing 300 µL LB medium with 50 µg/mL kanamycin, and the deep-well plates were cultured overnight at 37 °C with 800 rpm. DNA sequencing was performed, and the 19 diverse variants were obtained with the mutation site respectively replaced by the remaining AA residues. For protein expression, an overnight incubation was performed for the 19 variants in 5 mL LB-medium containing 50 µg/mL kanamycin with shaking at 37 °C, 220 rpm. An aliquot of 500 µL pre-culture was inoculated to a new culture flask containing 50 mL LB medium with 50 µg/mL kanamycin and to an OD_600_ value of 0.6~0.8. Isopropyl-*β*-*D*-thiogalactopyranoside (IPTG, 0.1 mM) was added to induce gene expression with the culture conditions at 20 °C, 200 rpm shaking for 18 h. The cells were harvested and washed twice with 100 mM potassium phosphate buffer (pH 7.0) and centrifuged at 5000 rpm for 30 min. The pellets were then resuspended in 5 mL 100 mM potassium phosphate buffer (pH 7.0) containing 6 U DNase I and 1 mg/mL lysozyme for breaking the cells at 30 °C, 200 rpm shaking for 2 h. Then, 500 µL stock solution containing NADP^+^ (1 mM), ketone **1** (30 mM, 1 eq), amine **a** (60 mM, 2eq), 1 mg/mL GDH, *D*-glucose (60 mM), and DMSO (10% *v*/*v*) was added. The reaction solutions were shaken at 30 °C, 200 rpm for 24 h and quenched by the addition of 0.1 mL saturated sodium carbonate. Then the reaction solutions were extracted with ethyl acetate (3 × 1.0 mL), and the combined organic extracts were dried using anhydrous sodium sulfate. Finally, the solvent was removed under reduced pressure and the extracts were dissolved in ethanol and subjected to HPLC analysis for measurement of the ee values.

### 3.5. Expression and Purification of IR36-M5 Protein

The plasmids containing the genes encoding target enzymes were transformed into *E. coli* BL21(DE3) competent cells for gene expression. Single colonies were picked up, and an overnight incubation was performed in LB-medium (10 mL) containing 50 µg/mL kanamycin overnight at 37 °C, 220 rpm to obtain the pre-cultures. The pre-culture (10 mL) was inoculated into 1 L LB-medium and the culture was incubated at 37 °C, with shaking at 220 rpm to an OD_600_ value of 0.6–0.8. Gene expression was induced by the addition of IPTG (0.1 mM), and incubation was continued at 20 °C, 200 rpm for 18 h. The cells were harvested by centrifugation at 5000 rpm for 30 min and resuspended in binding buffer (50 mM Tris-HCl buffer pH 8.0, 300 mM NaCl, containing 20 mM imidazole). Cells were disrupted by ultrasonication for 30 min (5 s on and 9 s off cycles). The suspension was centrifuged at 12,000 rpm for 25 min, and a clear lysate was obtained. The N-terminal His-tagged proteins were purified using the Ni-NTA column. In detail, the lysate was loaded onto a pre-equilibrated Ni-NTA column, followed by washing with 40~60 mL washing buffer (50 mM Tris-HCl buffer pH 8.0, 300 mM NaCl, containing 40 mM imidazole). The bound protein was eluted with an elution buffer containing 250 mM imidazole. The N-terminal His-tagged proteins were concentrated and used for further biotransformation reactions. 

### 3.6. Enzymic Reductive Aminations of IR36-M5 Proteins

We use purified IR36-M5 and its mutant proteins to measure the substrate loading. A typical 5 mL reaction mixture contained 10 mg/mL purified enzymes, 1 mM NADP^+^, 30 mM phenylacetone **1**, propargylamine **a** (2 eq), *D*-glucose (2 eq), 1 mg/mL GDH, and DMSO (10% *v*/*v*) in sodium phosphate buffer (100 mM, pH 7.0). All reactions were incubated at 30 °C with shaking at 220 rpm for 24 h, and then the reactions were quenched by the addition of 5 mL acetonitrile containing 1M acetic acid. Finally, the mixtures were centrifuged at 12,000 rpm for 10 min and the supernatant was subjected to HPLC analysis. 

### 3.7. Biotransformation Using Cell-Free Extract

Cell-free extracts of IR36-M5 and its mutant proteins were used to measure the conversion and enantioselectivity for ketone **1** and amine **a**. A typical 10 mL reaction mixture contained cell-free extract (10 g/L wet cells weight), 1 mM NADP^+^, 30 mM phenylacetone **1**, propargylamine **a** (2 eq, stock solution was adjusted to pH 7.0), *D*-glucose (2 eq), 1 mg/mL GDH, and DMSO (10% *v*/*v*) in sodium phosphate buffer (100 mM, pH 7.0). After being shaken for 24 h at 30 °C with 200 rpm, the reactions were quenched with acetic acid to adjust the solution to a pH of 3.0, and celite was added. The mixtures were then filtered and rinsed with water. The filtrate was extracted with EtOAc (3 × 10 mL) to remove neutral materials. The aqueous phase was alkalified to pH 10.0 with saturated sodium carbonate and extracted three times with 10 mL of dichloromethane. The solvent of the combined organic extract was removed under reduced pressure, and the corresponding amine products were finally obtained. 

### 3.8. HPLC and Chiral HPLC Analysis

HPLC analysis was performed on all the biotransformation products, and confirmation was conducted by comparing their UV spectra with standards. Substrate loading and conversions were calculated according to the standard curves of **1a**, which were plotted for varying concentrations of standards using the UV detection wavelength of 210 nm. The above-mentioned samples were analyzed by Waters 2695 Separation Module and Wates 2996 Photodiode Array Detector with gradient method (1.0 mL/min, 20 min, H_2_O/MeCN, 90/10→10/90, *v*/*v*) using C_18_ analytic column (Phenomenex Gemini, 4.6 × 250 mm, 5 μm, Phenomenex, Torrance, CA, USA). 

Chiral HPLC analysis was performed to analyze the ee values of racemic amine standards, chiral amine standards, and biotransformation products for **1a** with chiral columns and different solvent ratios of ethanol containing 0.02% diethylamine and *n*-hexane. 

### 3.9. Molecular Docking

In this study, the previously reported crystal structure of IR36-M5 (7WNW), which is a complex with NADP^+^, is used as the receptor target. The structures of M5 are generated by using the standard mutation protocol of Discovery Studio 2019 Client. Flexible docking of the imine intermediate of phenylacetone **1** and propargylamine **a** into M5 was performed using a flexible docking protocol. Docking runs were carried out using the standard parameters, and the conformations with the lowest energy were chosen for analysis of the substrate–enzyme interactions. 

## 4. Conclusions

In summary, we have identified five mutants (M203E, M203Q, F260M, H264N, and G268H), which displayed higher *R*-selectivity (98%, ee), although their conversion rates were low. Balancing both selectivity and conversion efficiency, the M5 mutant was chosen as the biocatalyst for synthesizing (*R*)-desmethylselegiline **1a**, a crucial intermediate for (*R*)-selegiline, a Parkinson’s disease therapy. The chemoenzymatic total synthesis of (*R*)-selegiline was successfully achieved in two steps, demonstrating good enantioselectivity (97%, *R*) and conversion (97%). This outcome highlights the potential of IR36-M5 in the reductive amination of homobenzylic ketones for producing chiral amines, emphasizing M5’s advantages in developing pharmaceuticals containing homobenzylic amines. Ongoing efforts in our lab focus on further optimizing M5 mutants to enhance stereoselectivity and conversion, with findings to be reported in due course.

## Figures and Tables

**Table 1:** Stereoselectivities of IRED mutants towards **1**.

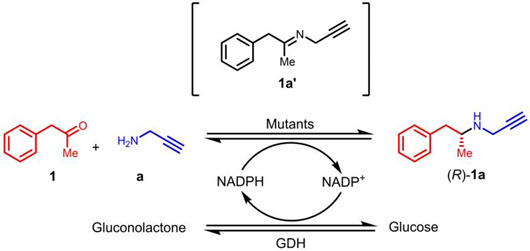			
IREDs	Substrate Loading (mM)	Enzyme Loading (mg/mL)	ee (%)
IR36-M5	30	10	97, *R*
M5-F260D	30	10	52, *R*
M5-F260E	30	10	76, *R*
M5-F260N	30	10	85, *R*
M5-W234L	30	10	76, *R*
M5-W234I	30	10	82, *R*

## Data Availability

Data are contained within the article and Supplementary Materials.

## Supplementary Materials

The following supporting information can be downloaded at https://www.mdpi.com/article/10.3390/molecules29061328/s1: Sequence of IR36-M5; Table S1: List of primers in this study; Table S2: Enantioselectivity of the mutants generated by site-saturation mutagenesis of residue M203 over M5; Table S3: Enantioselectivity of the mutants generated by site-saturation mutagenesis of residue F260 over M5; Table S4: Enantioselectivity of the mutants generated by site-saturation mutagenesis of residue H264 over M5; Table S5: Enantioselectivity of the mutants generated by site-saturation mutagenesis of residue G268 over M5; Table S6: Enantioselectivity of the mutants generated by site-saturation mutagenesis of residue L200 over M5; Figure S1: Enantioselectivity of IR36-M5 and its mutants at sites L200, respectively; Table S7: Enantioselectivity of the mutants generated by site-saturation mutagenesis of residue Y204 over M5; Figure S2: Enantioselectivity of IR36-M5 and its mutants at sites Y204, respectively; Table S8: Enantioselectivity of the mutants generated by site-saturation mutagenesis of residue W234 over M5; Figure S3: Enantioselectivity of IR36-M5 and its mutants at sites 234, respectively; Table S9: Conversion rates and stereoselectivities of IRED mutants towards **1a**; Figure S4: Chiral HPLC analysis of racemic standard of **1a** and chiral amine standards of **1a**, M5 catalytic product **1a**; Figure S5: The ^1^H NMR spectrum of **1a** in chloroform-*d*_4_ (400 MHz); Figure S6: The ^13^C NMR spectrum of **1a** in chloroform-*d*_4_ (100 MHz); Figure S7: The ^1^H NMR spectrum of (*R*)-Selegiline in chloroform-*d*_4_ (400 MHz); Figure S8: The ^13^C NMR spectrum of (*R*)-Selegiline in chloroform-*d*_4_ (100 MHz).
