# Supplementary material for: Chemoenzymatic Synthesis of Selegiline: An Imine Reductase-Catalyzed Approach
Source: Molecules. 2024 Mar 16;29(6):1328. doi: 10.3390/molecules29061328 (PMC10974447; doi:10.3390/molecules29061328)
Supplement: Supplementary file 1 [file molecules-29-01328-s001.zip › molecules-2919671-supplementary.pdf]

Chemoenzymatic Synthesis of Selegiline: An Imine Reductase-Catalyzed Approach

Yuliang Hu [1,2], Jinping Bao [2], Dongyu Tang [2], Shushan Gao [2,3], Fei Wang [1], Zhongtao Ding [1,2,*] and Chengsen Cui [2,3,*]

[1] College of Bioscience and Bioengineering, Jiangxi Agricultural University, Nanchang 330045, China; huyuliang98@163.com (Y.H.)
[2] Tianjin Institute of Industrial Biotechnology, Chinese Academy of Sciences, Tianjin 300308, China
[3] National Technology Innovation Center of Synthetic Biology, Tianjin 300308, China
* Correspondence: dzhongtaochina@163.com (Z.D.); cuichs@tib.cas.cn (C.C.); Tel./Fax: +86-22-2482-8742 (C.C.)

Table of Contents

1. Sequence of IR36-M5 .... **Error! Bookmark not defined.**

2. Table S1. List of primers in this study 4

3. Table S2. Enantioselectivity of the mutants generated by site-saturation mutagenesis of residue M203 over M5 4

4. Table S3. Enantioselectivity of the mutants generated by site-saturation mutagenesis of residue F260 over M5... 4

5. Table S4. Enantioselectivity of the mutants generated by site-saturation mutagenesis of residue H264 over M5 4

6. Table S5. Enantioselectivity of the mutants generated by site-saturation mutagenesis of residue G268 over M5 5

7. Table S6. Enantioselectivity of the mutants generated by site-saturation mutagenesis of residue L200 over M5 5

8. Figure S1. Enantioselectivity of IR36-M5 and its mutants at sites L200, respectively 5

9. Table S7. Enantioselectivity of the mutants generated by site-saturation mutagenesis of residue Y204 over M5 5

10. Figure S2. Enantioselectivity of IR36-M5 and its mutants at sites Y204, respectively 6

11. Table S8. Enantioselectivity of the mutants generated by site-saturation mutagenesis of residue W234 over M5 6

12. Figure S3. Enantioselectivity of IR36-M5 and its mutants at sites 234, respectively 6

13. Table S9. Conversion rates and stereoselectivities of IRED mutants towards **1a** 7

14. Figure S4. Chiral HPLC analysis of racemic standard of **1a**, and chiral amine standards of **1a**, M5 catalytic product **1a** 3

15. Figure S5. The ^{1}H NMR spectrum of **1a** in chloroform-d_4 (400 MHz) 9

16. Figure S6. The ^{13}C NMR spectrum of **1a** in chloroform-d_4 (100 MHz) 9

17. Figure S7. The ^{1}H NMR spectrum of **Selegiline** in chloroform-d_4 (400 MHz) 10

18. Figure S8. The ^{13}C NMR spectrum of **Selegiline** in chloroform-d_4 (100 MHz) ....**Error! Bookmark not defined.**

1. Sequence of IR36-M5

DNA sequence of IR36-M5

ATGGGCAGCAGCCATCATCATCATCATCACAGCAGCGGCCTGGTGCCGCGCGGCAGCCATATGCCGGA
ATCTACCACCCCGAGTACCGCCACCCCGGTGACCATCATCGGTCTTGGTGCAATGGGCACCGCCCTGG
CAAACGCATTCCTCGATGCAGGTCATAGTACCACCGTTTGGAATCGTACCGCAGCACGCGCCACCGCA
TTAGCCGCACGCGGCGCACATCATGCAGAAACCGTGACCGAAGCCATTGCAGCCTCTCCGTTAGTGAT
TGCCTGTGTGCTGGATTATGATGCCTTTCATGAAACCTTAGCCCCGGCTACAGACGCGCTGGCAGGTCG
CGCCCTGGTTAATCTGACCACAGGTACCCCGAAACAGGCACGCGAAACCGCCTCTTGGGCAGCCGAT
CATCGTATTGATTATCTGGATGGCAAAATTATGGCCATTCCGCCGGGTATTGCAACCCCGGATAGTTTTA
TTCTGTATAGCGGTCCGTTAGGTACCTTTGAAGCACATCGCTCAACCTTAGAAGTGCTGGGCGCAGCA
AATCATGTGGGTACCGATGCAGGTTTGGCGAGCTTACATGATATTGCACTGCTGACCGGTATGTATGGC
ATGATTGCAGGCATTTTACAGGCCTTTGCCTTAATTGATAGTGAAGGTATTCCGGCAGGCGATCTGGCC
CCGATGTTAACCAATTGGTTAACCGGCGCAGCACATAGCGTGGCCCATTATGCCCAGCAGATTGATACC
GGCGATTATGAAACCGGTGTTGTGTTTAATTTAGCACATCAGAGCCATGGCTTTGCAAAATTAGTTCAG
GCCGGTGAAGATCAGGGTGTGGATGTGGGCTTACTGCGTCCGCTGTTTGAACTGATGCGTCATCAGGT
TGCCGCAGGCTATGGTAATGGTGATGTTGCCTCAGTTATTGAACTGATTCGTCGCGAAGAACGTCGTCA
GCCGGCCAAAAGTCCGGGCGCAGATAAAATTACCCGTGCACGTCGTCCGTAA

Amino acid sequence of IR36-M5

MGSSHHHHHHSSGLVPRGSHMPESTTPSTATPVTIIGLGAMGTALANAFLDAGHSTTVWNRTAARATALA
ARGAHHAETVTEAIAASPLVIACVLDYDAFHETLAPATDALAGRALVNLTTGTPKQARETASWAADHRID
YLDGKIMAIPPGIATPDSFILYSGPLGTFEAHRSTLEVLGAANHVGTDAGLASLHDIALLTGMYGMIAGILQ
AFALIDSEGIPAGDLAPMLTNWLTGAAHSVAHYAQQIDTGDYETGVVFNLAHQSHGFAKLVQAGEDQGV
DVGLLRPLFELMRHQVAAGYGNGDVASVIELIRREERRQPAKSPGADKITRARRP*

Table S1. List of primers in this study.

Primer	mutant	Sequences (5′→3′)
F	L200NNK	ACCGGTTNNKAGTGCAATATCATGTAAGCTCGCCA
R	L200NNK	TATTGCACTMNNAACCGGTATGTATGGCATGATTG
F	M203NNK	GCCTGCAATCATGCCATAMNNACCGGTCAGCAGTG
R	M203NNK	NNKTATGGCATGATTGCAGGCATTTTACAGGCCTT
F	Y204NNK	AATGCCTGCAATCATGCCMNNCATACCGGTCAGCA
R	Y204NNK	NNKGGCATGATTGCAGGCATTTTACAGGCCTTTGC
F	W234NNK	ACCAATNNKTTAACCGGCGCAGCACATAGC
R	W234NNK	GCCGGTTAAMNNATTGGTTAACATCGGGGC
F	F260NNK	GTTGTGNNKAATTTAGCACATCAGAG
R	F260NNK	CTAAATTMNNCACAACACCGGTTTCA
F	H264NNK	TGCAAAGCCATGGCTCTGMNNTGCTAAATTAAACA
R	H264NNK	NNKCAGAGCCATGGCTTTGCAAAATTAGTTCAGGC
F	G268NNK	CTGAACTAATTTTGCAAAMNNATGGCTCTGATGTG
R	G268NNK	NNKTTTGCAAAATTAGTTCAGGCCGGTGAAGATCA

Table S2. Enantioselectivity of the mutants generated by site-saturation mutagenesis of residue M203 over M5.

Mutants	ee value	Mutants	ee value	Mutants	ee value
M203A	92%, *R*	M203I	N.D.	M203S	91%, *R*
M203C	86%, *R*	M203K	29%, *R*	M203T	70%, *R*
M203D	N.D.	M203L	96%, *R*	M203V	85%, *R*
M203E	98%, R	M203N	79%, *R*	M203W	63%, *R*
M203F	70%, *R*	M203P	89%, *R*	M203Y	66%, *R*
M203G	N.D.	M203Q	98%, R		
M203H	82%, *R*	M203R	97%, *R*		

Table S3. Enantioselectivity of the mutants generated by site-saturation mutagenesis of residue F260 over M5.

Mutants	ee value	Mutants	ee value	Mutants	ee value
F260A	85%, *R*	F260K	N.D.	F260S	92%, *R*
F260C	88%, *R*	F260L	77%, *R*	F260T	87%, *R*
F260D	52%, *R*	F260M	98%, R	F260V	67%, *R*
F260E	76%, *R*	F260N	85%, *R*	F260W	93%, *R*
F260G	96%, *R*	F260P	52%, *R*	F260Y	95%, *R*
F260H	93%, *R*	F260Q	N.D.		
F260I	27%, *R*	F260R	N.D.		

Table S4. Enantioselectivity of the mutants generated by site-saturation mutagenesis of residue H264 over M5.

Mutants	ee value	Mutants	ee value	Mutants	ee value
H264A	96%, *R*	H264K	96%, *R*	H264S	97%, *R*
H264C	95%, *R*	H264L	95%, *R*	H264T	92%, *R*
H264D	94%, *R*	H264M	96%, *R*	H264V	83%, *R*
H264E	96%, *R*	H264N	98%, R	H264W	60%, *R*
H264F	83%, *R*	H264P	94%, *R*	H264Y	94%, *R*
H264G	97%, *R*	H264Q	96%, *R*		
H264I	93%, *R*	H264R	94%, *R*		

Table S5. Enantioselectivity of the mutants generated by site-saturation mutagenesis of residue G268 over M5.

Mutants	ee value	Mutants	ee value	Mutants	ee value
G268A	86%, *R*	G268K	N.D.	G268S	96%, *R*
G268C	71%, *R*	G268L	16%, *R*	G268T	44%, *R*
G268D	76%, *R*	G268M	92%, *R*	G268V	23%, *R*
G268E	92%, *R*	G268N	87%, *R*	G268W	94%, *R*
G268F	97%, *R*	G268P	83%, *R*	G268Y	53%, *R*
G268H	98%, *R*	G268Q	97%, *R*		
G268I	58%, *R*	G268R	N.D.		

Table S6. Enantioselectivity of the mutants generated by site-saturation mutagenesis of residue L200 over M5.

Mutants	ee value	Mutants	ee value	Mutants	ee value
L200A	57%, *R*	L200I	86%, *R*	L200S	39%, *R*
L200C	51%, *R*	L200K	N.D.	L200T	35%, *R*
L200D	37%, *R*	L200M	95%, *R*	L200V	86%, *R*
L200E	41%, *R*	L200N	89%, *R*	L200W	N.D.
L200F	58%, *R*	L200P	N.D.	L200Y	N.D.
L200G	79%, *R*	L200Q	47%, *R*		
L200H	N.D.	L200R	N.D.		

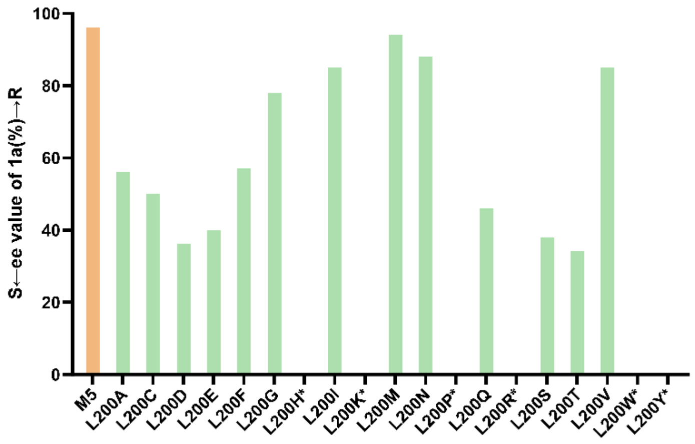

Figure S1. Enantioselectivity of IR36-M5 and its mutants at sites L200, respectively.

Table S7. Enantioselectivity of the mutants generated by site-saturation mutagenesis of residue Y204 over M5.

Mutants	ee value	Mutants	ee value	Mutants	ee value
Y204A	72%, *R*	Y204I	55%, *R*	Y204R	N.D.
Y204C	62%, *R*	Y204K	N.D.	Y204S	72%, *R*
Y204D	88%, *R*	Y204L	48%, *R*	Y204T	76%, *R*
Y204E	89%, *R*	Y204M	89%, *R*	Y204V	62%, *R*
Y204F	N.D.	Y204N	94%, *R*	Y204W	94%, *R*
Y204G	64%, *R*	Y204P	N.D.		
Y204H	90%, *R*	Y204Q	88%, *R*		

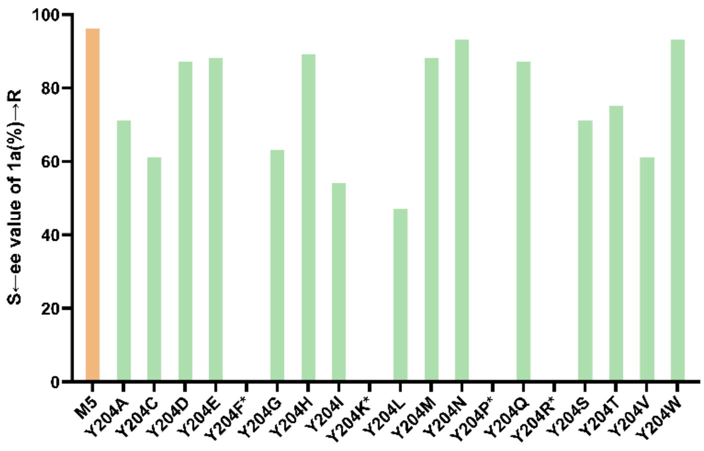

Figure S2. Enantioselectivity of IR36-M5 and its mutants at sites Y204, respectively.

Table S8. Enantioselectivity of the mutants generated by site-saturation mutagenesis of residue W234 over M5.

Mutants	ee value	Mutants	ee value	Mutants	ee value
W234A	86%, *R*	W234I	82%, *R*	W234R	95%, *R*
W234C	89%, *R*	W234K	86%, *R*	W234S	89%, *R*
W234D	88%, *R*	W234L	76%, *R*	W234T	89%, *R*
W234E	51%, *R*	W234M	78%, *R*	W234V	92%, *R*
W234F	93%, *R*	W234N	85%, *R*	W234Y	96%, *R*
W234G	79%, *R*	W234P	92%, *R*		
W234H	91%, *R*	W234Q	84%, *R*		

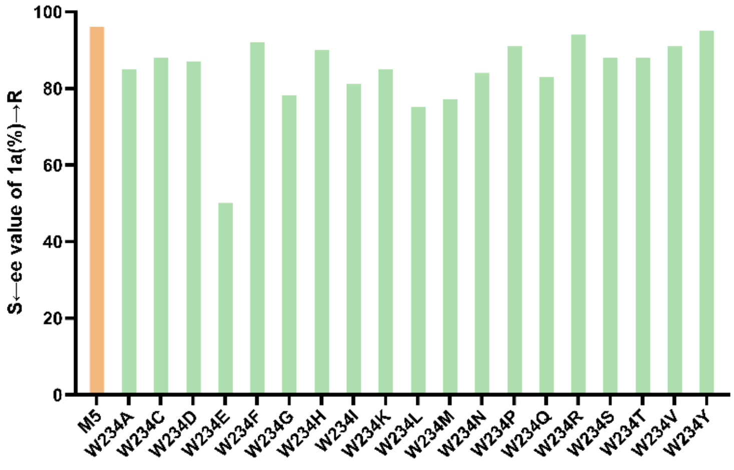

Figure S3. Enantioselectivity of IR36-M5 and its mutants at sites 234, respectively.

Table S9. Conversion rates and stereoselectivities of IRED mutants towards **1a**.

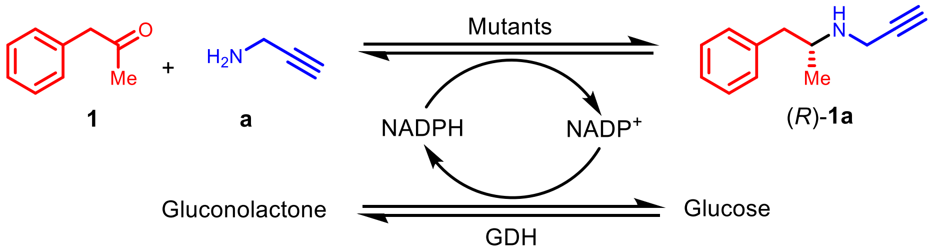

**Entry**	**Mutants**	**Substrate loading (mM)**	**Enzyme loading (mg mL^{-1})**	***Conv.(%)***	***ee* (%)**
1	IR36-M5	30	10	97	97, *R*
2	M5-M203E	30	10	34	98, *R*
3	M5-M203Q	30	10	42	98, *R*
4	M5-F260M	30	10	9	98, *R*
5	M5-H264N	30	10	58	98, *R*
6	M5-G268H	30	10	65	98, *R*

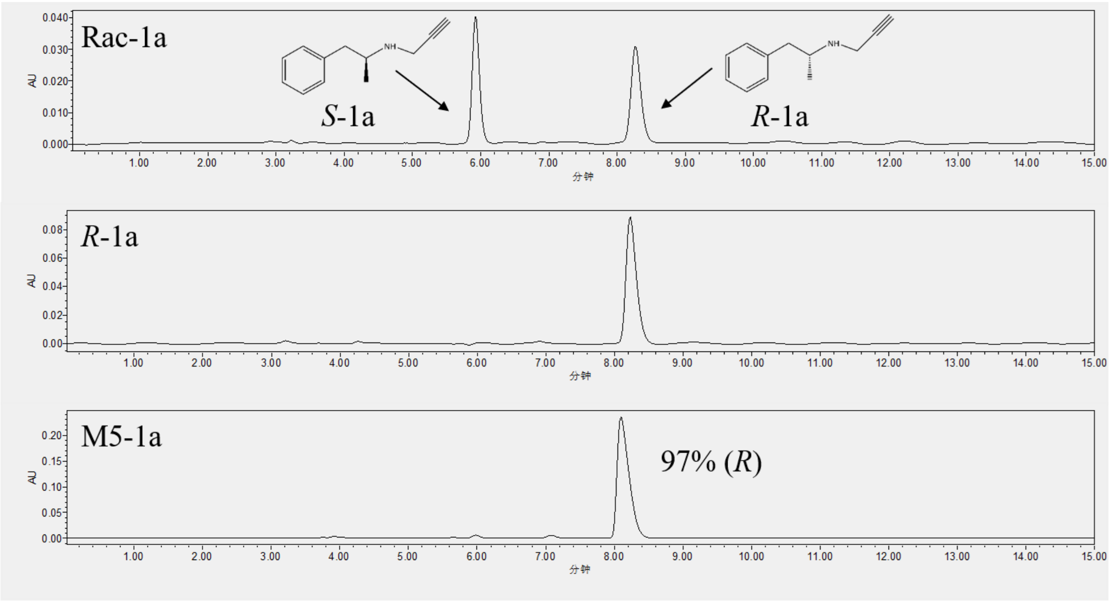

Figure S4. Chiral HPLC analysis of racemic standard of **1a**, and chiral amine standards of **1a**, M5 catalytic product **1a**.

HPLC conditions: CHIRALPAK IG column with a mobile phase of *n*-hexane/ethanol (90:10, v/v, 0.2‰ diethylamine), flow rate 1.0 mL/min, 30 °C, UV detection at 258.4 nm.

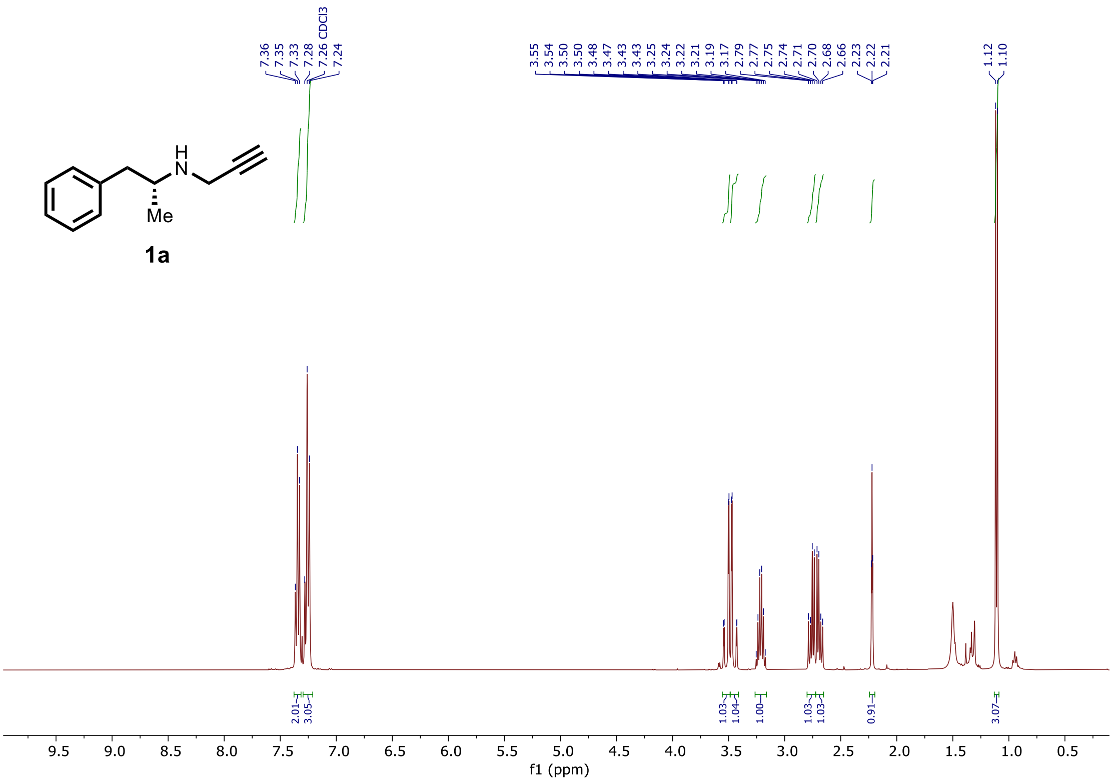

Figure S5. The ^{1}H NMR spectrum of **1a** in chloroform-d_4 (400 MHz)

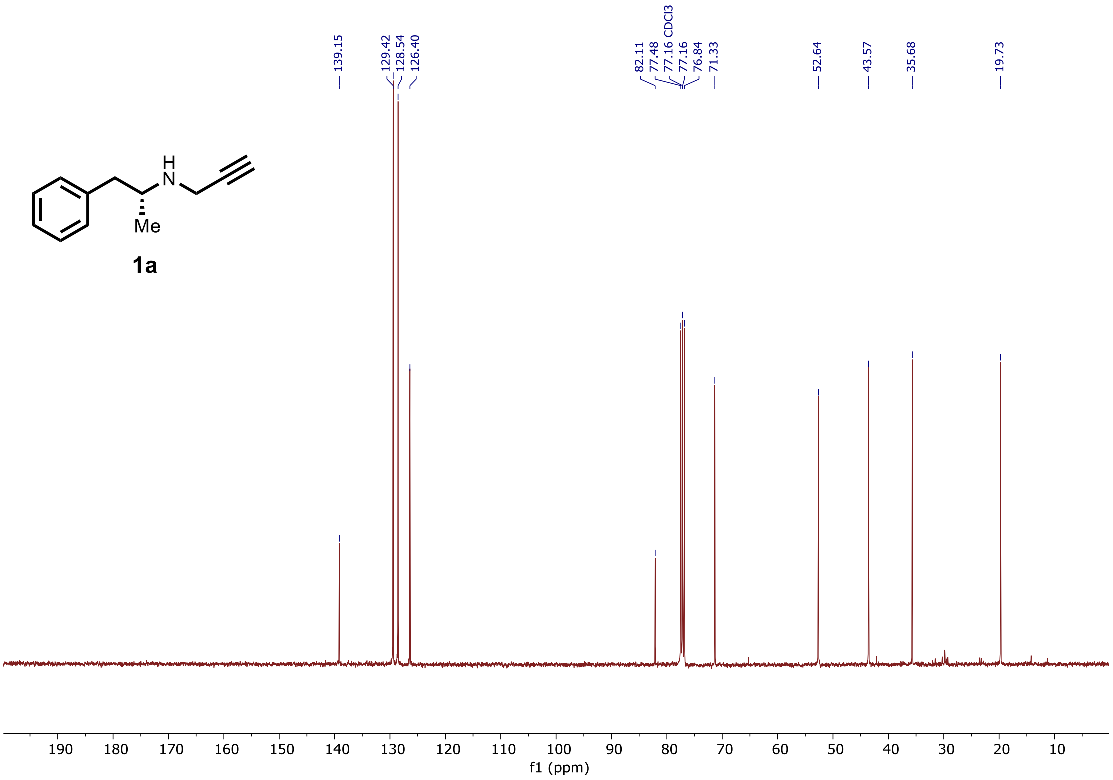

Figure S6. The ^{13}C NMR spectrum of **1a** in chloroform-d_4 (100 MHz)

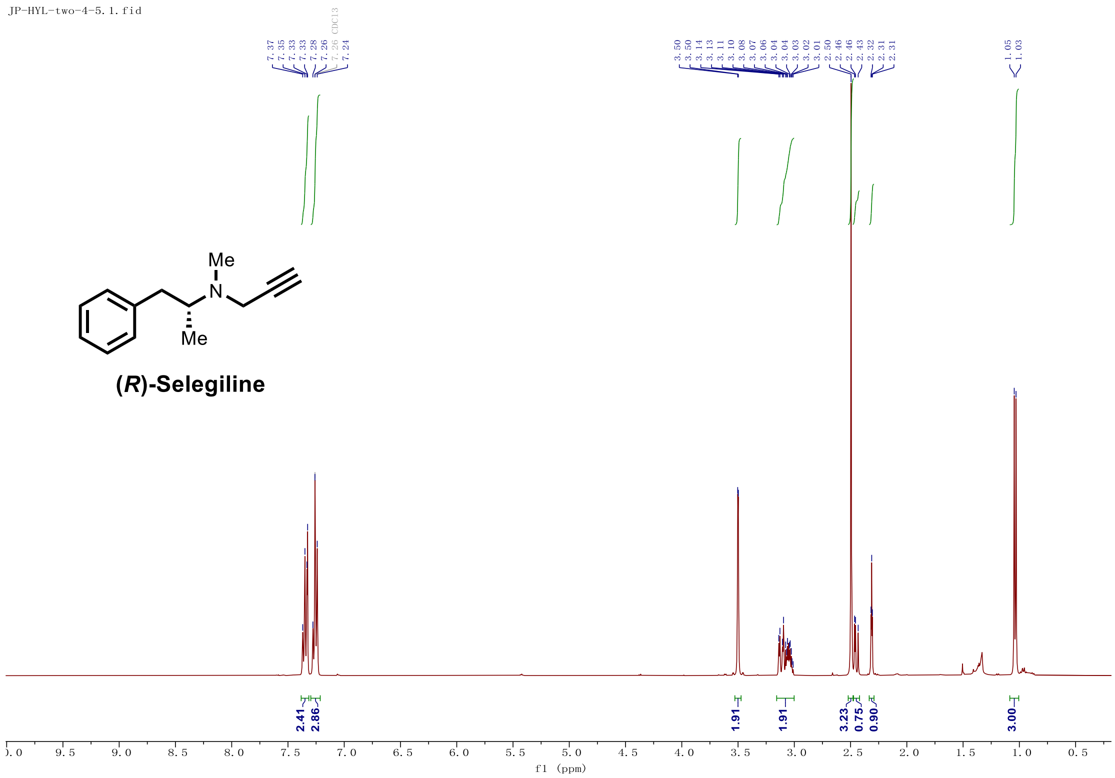

Figure S7. The ¹H NMR spectrum of **Selegiline** in chloroform-d_4 (400 MHz)

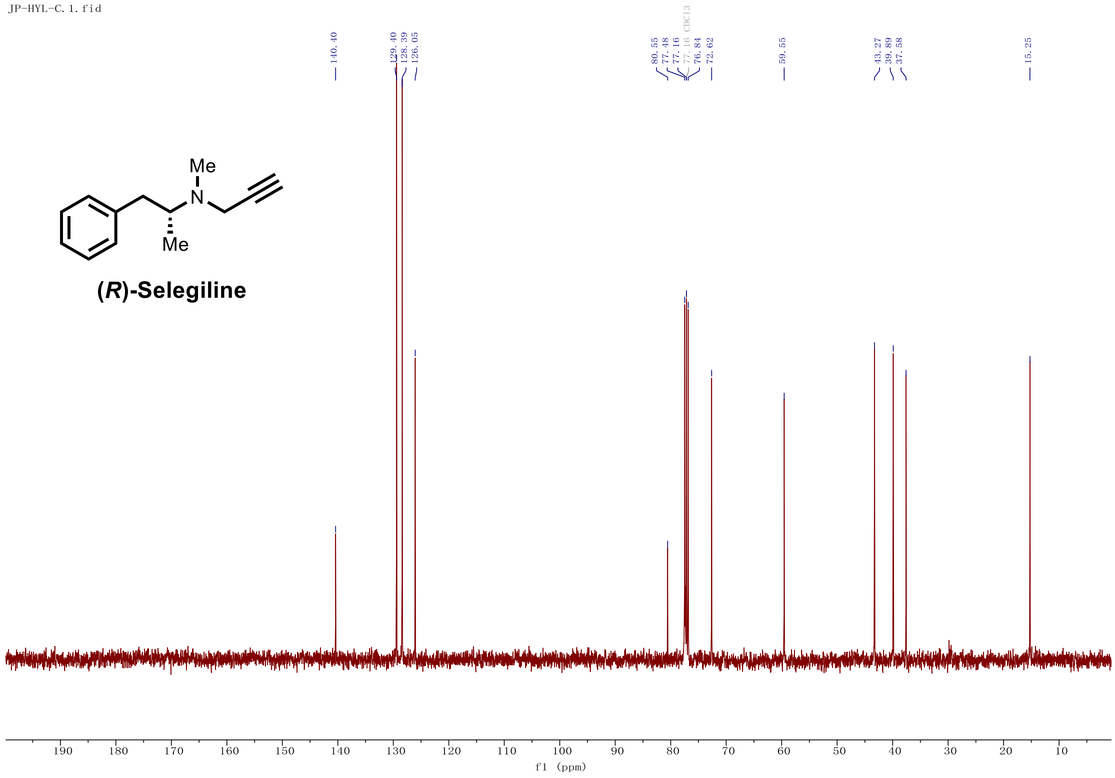

Figure S8. The ^{13}C NMR spectrum of **Selegiline** in chloroform-d_4 (100 MHz)
